# Estrobolome dysbiosis in canine mammary gland tumors: *Eggerthella lenta* as a candidate microbial marker for hormone-dependent mammary tumorigenesis

**DOI:** 10.3389/fmicb.2026.1923235

**Published:** 2026-09-02

**Authors:** Kun-Woo Kim, Mahnoor Farooq, Do Young Jin, Wonhong Kim, Jiho Shin, Suji Park, Ginseo Ko, Seohyeon Park, Gyeongtae Kim, Seunghyun Na, Minsoo Kim, Yae-ji Kim, Chaehyun Kim, Ho Jun Lee, Sungyoung Choi, Hyo-Jin Ahn, Hyuk-Ju Jang, Han-Sol Choi, Ki-Young Yoon, Kyung-Ku Kang, Suk-Chae Jung, Woo Jin Song, Jun-Seob Kim, Young Hyun Jung

**Affiliations:** 1 Laboratory of Veterinary Internal Medicine, College of Veterinary Medicine, Jeju National University, Jeju, Republic of Korea; 2 FM Animal Medical Center, Goyang-si, Gyeonggi-do, Republic of Korea; 3 Department of Physiology, College of Medicine, Soonchunhyang University, Cheonan, Republic of Korea; 4 Institute for Molecular Metabolism Innovation, Soonchunhyang University, Asan, Republic of Korea; 5 Department of Nano-Bioengineering, Incheon National University, Incheon, Republic of Korea; 6 New S Animal Hospital, Gunpo-si, Gyeonggi-do, Republic of Korea; 7 Able Animal Medical Center, Seocho-gu, Seoul, Republic of Korea; 8 Department of Veterinary Nursing, Shingu College, Seongnam-si, Gyeonggi-do, Republic of Korea; 9 Department of Companion Animal, Shingu College, Seongnam-si, Gyeonggi-do, Republic of Korea; 10 Preclinical Research Center, K-MEDI hub, Daegu, Republic of Korea; 11 Department of Chemical and Biomolecular Engineering, University of Illinois at Urbana-Champaign, Urbana, IL, United States; 12 Signature Animal Medical Center, Seoul, Republic of Korea

**Keywords:** 16S rRNA, canine mammary gland tumor, *Eggerthella lenta*, estrobolome, gut microbiome, β-glucuronidase

## Abstract

Canine mammary gland tumors (MGTs) are the most prevalent neoplasms in intact female dogs, yet the role of the gut microbiome in their pathogenesis remains largely unexplored. This pilot study investigated the gut microbiota composition of 40 female dogs classified into MGT (*n* = 11), Non-MGT (*n* = 20), and under 2 years (U2Y, *n* = 9) groups using 16S rRNA gene sequencing. Alpha diversity indices showed no significant differences between the MGT and Non-MGT groups, whereas beta diversity analysis revealed significant overall community-level differences across all three groups (PERMANOVA, *p* = 0.0001), with the U2Y group compositionally distinct from both adult groups. In contrast, MGT and Non-MGT groups showed substantial overlap in community structure after correction for multiple comparisons. Differential abundance analysis identified *Eggerthella* as a candidate MGT-associated taxon, and species-specific quantitative PCR (qPCR) demonstrated a significantly higher normalized abundance of *Eggerthella lenta* in MGT samples than in Non-MGT controls (Mann–Whitney *U* test, *p* = 0.0159). Conversely, potentially beneficial short-chain fatty acid (SCFA)-producing bacteria, including *Clostridium* and *Megamonas*, showed reduced relative abundance in tumor-bearing dogs (*Clostridium*, ANCOM-BC; *Megamonas*, exploratory *post hoc* Mann–Whitney test). PICRUSt2 analysis suggested a trend toward enrichment of polyamine biosynthesis pathways in the MGT group, although no pathway remained significant after multiple-testing correction in the MGT vs. Non-MGT comparison. To our knowledge, this study provides the first evidence linking the estrobolome-associated bacterium *Eggerthella lenta* with canine MGTs. Together with the depletion of beneficial SCFA-producing bacteria, these findings suggest a pattern of gut microbial dysbiosis that may influence hormone-dependent mammary tumorigenesis through microbiome-associated alterations in estrogen metabolism. Although *E. lenta* was detected in both MGT and Non-MGT dogs, its significantly higher normalized abundance in MGT samples supports its potential as an abundance-based candidate microbial marker that warrants validation in large, independent prospective cohorts before clinical application. Collectively, these findings provide new insights into the gut–mammary axis and support future investigations into microbiome-based diagnostic and therapeutic strategies for canine mammary gland tumors.

## Introduction

Canine mammary gland tumors (MGTs) are considered the most common tumors affecting intact female dogs, representing approximately 40%−50% of all neoplasms in this population, with some studies reporting prevalence rates of up to 70% (Yoshida et al., 2014; Osaki et al., 2016; Zheng et al., 2022b). Approximately half of these tumors are malignant and have the potential to metastasize, which makes them a major challenge in veterinary oncology (Yoshida et al., 2014; Zheng et al., 2022b). Despite their high prevalence, the precise genetic and molecular mechanisms driving mammary tumorigenesis in dogs remain incompletely understood. Notably, spayed dogs have less than a 0.5% chance of developing MGTs, which provides compelling evidence that hormonal signaling is central to tumor development (Nosalova et al., 2024; da Silva et al., 2023; Vazquez et al., 2023).

Estrogen, among other hormonal factors, plays a central role in the pathogenesis of canine MGTs by stimulating mammary glandular cell proliferation, inducing DNA damage, and regulating important signaling pathways that govern tumor growth and metastasis (Kabir et al., 2016; Torres et al., 2021; Ke et al., 2024). Approximately 88% of malignant canine mammary tumors are hormone receptor-positive, highlighting the critical importance of estrogen signaling in this malignancy (Gautam et al., 2025). Estrogen-driven mitogenesis further intersects with the regulation of angiogenesis and the tumor microenvironment, reinforcing the clinical relevance of hormone receptor status in both diagnosis and treatment (Abdelmegeed and Mohammed, 2018; Torres et al., 2021; Valdivia et al., 2021; Vazquez et al., 2023).

Early diagnosis of MGT is crucial given the high malignancy rate and poor prognosis associated with advanced disease (Valdivia et al., 2021). Current diagnostic approaches rely on clinical examination, fine needle aspiration, and histopathological evaluation of biopsy tissue which may be invasive and may not detect early-stage MGTs (Sontas et al., 2012; Kaszak et al., 2022; Pakdeesaneha et al., 2024). Conventional hematological and biochemical tests are often insufficient to detect systemic manifestations of early and benign MGTs (Nosalova et al., 2024). Therefore there is a need for a non-intrusive test for diagnosing early-stage MGTs and providing an accurate prognosis which would allow timely therapeutic intervention thereby improving the survival rates of affected individuals.

The gut microbiome is a collection of trillions of microorganisms that populate the gastrointestinal tract and is increasingly being recognized as a key regulator of host physiology and cancer biology (Cao et al., 2024; Filippo et al., 2024; Mishra and Mishra, 2024). Microbiome dysbiosis has been associated with the development of cancer in both humans and animal tumor models through systemic inflammation changes in host metabolism and microbial metabolite production (Zheng et al., 2022a; Aluai-Cunha et al., 2023; Breczko et al., 2024; Santiago-Rodriguez, 2024). The estrobolome refers to the collection of gut microorganisms and their associated genes that are capable of metabolizing estrogens, thereby regulating systemic estrogen homeostasis through enterohepatic circulation (Plottel and Blaser, 2011; Kwa et al., 2016; Baker et al., 2017; Larnder et al., 2025; Tahri and Amedei, 2025). Many members of the gut microbiota express β-glucuronidase (encoded by the gus/uidA gene family), an enzyme that deconjugates estrogen glucuronides in the intestine, allowing biologically active estrogens to be reabsorbed into the circulation (Yokoyama and Suzuki, 2008; Baker et al., 2017; Ervin et al., 2019; Hu et al., 2023). Through this regulation of estrogen bioavailability, the estrobolome has emerged as an important contributor to hormone-dependent cancers, particularly breast cancer, where altered microbial composition and increased β-glucuronidase activity have been associated with elevated circulating estrogen levels, tumor progression, and disease risk (Plottel and Blaser, 2011; Ervin et al., 2019; Parida and Sharma, 2019; Tsai et al., 2025).

Although a recent study documented altered gut microbiome composition in dogs with MGTs (Zheng et al., 2022a), the specific involvement of estrobolome bacteria in canine mammary tumorigenesis has not previously been investigated. Among estrobolome-associated bacteria, *Eggerthella lenta* has attracted increasing attention because it possesses β-glucuronidase activity, enabling the deconjugation of estrogen metabolites and potentially influencing systemic estrogen availability (Baker et al., 2017; Ervin et al., 2019; Larnder et al., 2025). In addition, *E. lenta* has been reported to be enriched in several hormone-related and gastrointestinal diseases and has been implicated in host metabolic and immune regulation, suggesting that it may play a broader role in disease pathogenesis (Koppel et al., 2017; Alexander et al., 2022). Given its established role in estrogen metabolism and its reported association with hormone-related diseases, *E. lenta* represents a biologically plausible candidate for investigating microbiome–host interactions in canine MGTs. However, its potential role in canine MGTs remains unknown. Characterizing estrobolome-associated bacteria, including *E. lenta*, may therefore provide meaningful insights into the gut–mammary axis in hormone-dependent cancer and open new avenues for non-invasive microbiome-based diagnostics (Veziant et al., 2021; Santiago-Rodriguez, 2024).

We hypothesized that gut microbial dysbiosis, particularly involving estrobolome bacteria with β-glucuronidase activity, plays a role in the pathogenesis of MGTs, with specific microbial alterations differentiating between affected dogs and controls without mammary tumors. The present study aimed to determine the gut microbiome profile of female canines with MGTs and compare it with age-matched controls without mammary tumors. An additional cohort of young dogs (under 2 years) was included to distinguish tumor-associated microbial changes from age-related variations. This study further investigated predicted bacterial functional pathways, including those involved in estrogen metabolism, using PICRUSt2 based on 16S rRNA sequencing data. Through this comprehensive analysis, we aimed to identify gut microbial signatures, candidate microbial markers, and predicted functional alterations associated with canine MGTs that may contribute to early disease detection and improve our understanding of the gut–mammary axis.

## Methods

### Sample collection and cohort stratification

**Figure 1:**
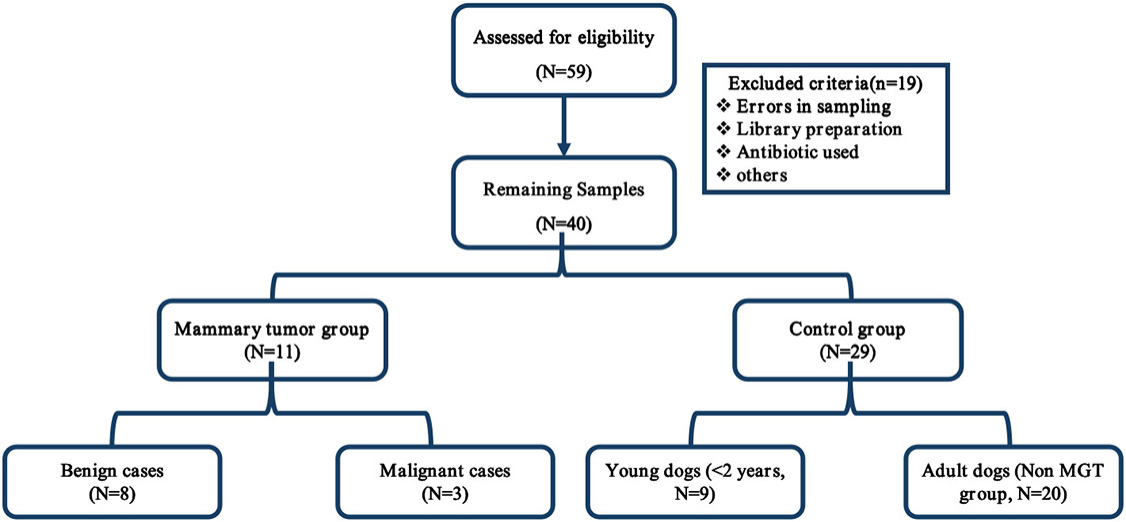
Flowchart of study subject enrollment and group classification. A total of 59 subjects were initially assessed for eligibility. Nineteen subjects were excluded based on specific criteria, including errors in sampling, library preparation issues, and antibiotic usage. The remaining 40 samples were divided into the Mammary tumor group (*n* = 11) and the control group (*n* = 29). The Mammary tumor group comprised benign (*n* = 8) and malignant (*n* = 3) cases. The control group was stratified by age into Young Dogs ( ≤ 2 years, *n* = 9; U2Y group) and adult dogs (*n* = 20; Non-MGT group).

The Institutional Animal Care and Use Committee (IACUC) of Soonchunhyang University (approval number: SCH24-0058) approved the use of fecal samples obtained from dogs for research purposes. Canine fecal samples were collected from FM Animal Medical Center, Goyang-si, and all owners provided written informed consent prior to enrollment. Of an initial 59 canine fecal samples, 19 were excluded due to sampling errors, suboptimal library preparation, inconsistent signalment data, or recent antibiotic administration. The remaining 40 samples were included in the final analysis. The cohort was stratified into three groups: the MGT group (*n* = 11), comprising dogs with histopathologically confirmed mammary gland tumors classified as benign (*n* = 8) or malignant (*n* = 3) based on standard WHO histological criteria for canine mammary tumors; the Non-MGT group (*n* = 20), comprising age-matched healthy female dogs without clinical or histopathological evidence of mammary neoplasia, although some individuals presented with non-neoplastic comorbidities as detailed in Table 1; and the U2Y group (*n* = 9), comprising healthy female dogs under 2 years of age (Figure 1). This three-group design enabled tumor-associated microbiota changes to be distinguished from those attributable to normal age-related variation in microbial community composition.

**Table 1:** Details of Non-MGT group dogs.

Sex/Age	Breed	Diagnosis
SF/7 years	Mix	IVDV
SF/7 years	Poodle	High LFT
SF/5 years	Bichon Frise	
SF/10 years	Shih Tzu	
SF/9 years	Yorkshire Terrier	
SF/13 years	Maltese	
SF/8 years	Poodle	
SF/9 years	Chihuahua	
SF/8 years	Poodle	
SF/9 years	Chihuahua	
F/9 years	Mix	
SF/14 years	Maltese	
SF/11 years	Maltese	
F/10 years	Mix	
SF/10 years	Maltese	
SF/14 years	Maltese	
SF/12 years	Yorkshire Terrier	
SF/10 years	West Highland White Terrier	
SF/10 years	Coton de Tulear	
SF/10 years	Yorkshire Terrier	

### DNA extraction and 16S rRNA library preparation

Fecal DNA was extracted using the QIAsymphony^®^ PowerFecal^®^ Pro DNA Kit (Qiagen, Hilden, Germany) in accordance with the manufacturer's protocol. Extracted DNA was assessed for quality and quantity prior to amplicon library preparation. The V3–V4 hypervariable region of the 16S rRNA gene was amplified using the established primer pair 341F (5′-CCTACGGGNGGCWGCAG−3′) and 805R (5′-GACTACHVGGGTATCTAATCC−3′) (Huang et al., 2025).

Amplicon quality was assessed using an Agilent 5400 Fragment Analyzer (Agilent Technologies, Santa Clara, CA, USA), an automated capillary electrophoresis system that evaluates fragment size distribution and DNA concentration using a fluorescence-based detection system with an integrated capillary array. This quality control step confirmed the absence of non-specific amplification, primer-dimer formation, and sample degradation in all 59 samples prior to sequencing. Validated amplicons were sequenced on the NovaSeq™ platform (Illumina, San Diego, CA, USA) using paired-end 2 × 250 bp read configuration.

### Sequencing data quality control

Raw sequencing quality was assessed using Phred quality scoring, where the base quality score = −10log10(e), with e representing the per-base sequencing error rate. All samples were required to maintain quality scores predominantly above Q20 (error rate ≤ 1%) to ensure reliable downstream analysis. Following quality assessment, raw reads underwent filtering based on a sliding window quality threshold (minimum quality score of 30 per five-base window), removal of adapter and barcode sequences, and chimera checking. Clean reads passing all quality filters were retained for bioinformatic analysis.

### Bioinformatic processing and taxonomic classification

All 59 collected samples underwent sequencing; the 19 subsequently excluded samples were removed after sequencing based on quality thresholds and signalment criteria applied during post-sequencing review. The remaining 40 high-quality samples were retained for all downstream analyses. All 59 raw 16S rRNA sequencing samples were initially processed using QIIME2 (version 2024.5; Northern Arizona University, Flagstaff, AZ, USA), (Bolyen et al., 2019). Paired-end reads were imported, and primer sequences were removed. Denoising, chimera removal, and amplicon sequence variant (ASV) generation were performed using the DADA2 algorithm (Callahan et al., 2016). Taxonomic classification of ASVs was performed against the SILVA 138.2 database (Quast et al., 2013). All downstream diversity calculations and statistical comparisons were also performed within the QIIME2 framework. Visualization and figure generation were carried out using R.

### Diversity analysis

Alpha diversity was evaluated using two complementary indices: Observed Features and the Shannon Diversity Index, to evaluate species richness and overall community diversity. Group-level differences in alpha diversity were assessed using the Kruskal–Wallis test, with results visualized using violin plots. *p*-Values were adjusted for multiple comparisons using the Benjamini–Hochberg method to obtain *q*-values.

Beta diversity was assessed using four distance metrics: the Bray–Curtis dissimilarity, Jaccard index, unweighted UniFrac, and weighted UniFrac distance. Between-group microbial community differences were visualized using principal coordinate analysis (PCoA) plots. Overall statistical significance of group separation was assessed using PERMANOVA (9,999 permutations), and *post-hoc* pairwise comparisons between groups were performed using pairwise PERMANOVA with 9,999 permutations, and *p*-values were adjusted using the Benjamini–Hochberg method.

### PICRUSt2 analysis

Functional pathway prediction was conducted using PICRUSt2 (version 2.5.3; Dalhousie University, Halifax, NS, Canada). ASV representative sequences and a feature table containing ASV counts, and taxonomy data were phylogenetically placed into a reference tree using place_seqs.py (Douglas et al., 2020). The sequence placement outputs were then used to perform hidden state prediction via hsp.py, which inferred the functional potential of the metagenome by predicting gene family abundances (e.g., Enzyme Commission numbers and KEGG Orthologs). Finally, pathway-level abundances were predicted using pathway_pipeline.py, followed by annotation with descriptions from the MetaCyc database.

### Species-specific qPCR validation

*Eggerthella lenta*–specific primers were designed using the Phylogenetically Unique Primers in Python (PUPpy) platform to ensure species-level specificity. Coding sequence (CDS) files were aligned using MMseqs2, and primer pairs were selected on the basis of low pair penalty scores, 40%−60% GC content and melting temperatures between 55 and 60 °C. Primer specificity was confirmed *in silico* against the NCBI nucleotide database using BLAST. The final validated primer sequences for *E. lenta* were: Forward 5′-ACCCTGTTGAGCGTGTATCC−3′ and Reverse 5′-ATCGACTCCGCCGTATTCTG−3′.

Quantitative PCR was performed on fecal DNA extracted from MGT (*n* = 11) and Non-MGT (*n* = 20) samples. Reactions were carried out in a 20 μL volume containing 10 μL of 2 × SYBR Green PCR Master Mix, 0.4 μM of each primer, and 2 μL of template DNA. Cycling conditions comprised an initial denaturation at 95 °C for 3 min, followed by 60 cycles of denaturation at 95 °C for 10 s, annealing at 55 °C for 10 s, and extension at 72 °C for 30 s with a final melt curve analysis to confirm amplification specificity (Supplementary Figure S1). Species-specific qPCR was used to estimate the relative abundance of *E. lenta* DNA rather than its simple presence or absence. Cycle threshold (Ct) values were recorded, and relative abundance was calculated using the ΔΔCt method after normalization to the universal bacterial 16S rRNA reference gene.

### Statistical analysis

Statistical analyses were performed using R software (version 4.4.3; R Foundation for Statistical Computing, Vienna, Austria). Continuous variables were compared between groups using Student's *t*-test or Mann-Whitney *U* test as appropriate. Categorical variables were analyzed using chi-square or Fisher's exact test. For microbiome analyses, PERMANOVA was used for beta diversity comparisons, and ANCOM-BC (R package ANCOMBC version 2.14.0) was used for differential abundance analysis. For taxa not identified by ANCOM-BC, exploratory *post-hoc* Mann–Whitney *U* tests were performed on relative abundance data, with *p* < 0.05 as a nominal threshold; these findings are considered secondary and hypothesis-generating. A *p*-value < 0.05 was considered statistically significant.

## Results

### Histopathological examination and clinical profiling of MGT and Non-MGT groups

**Figure 2:**
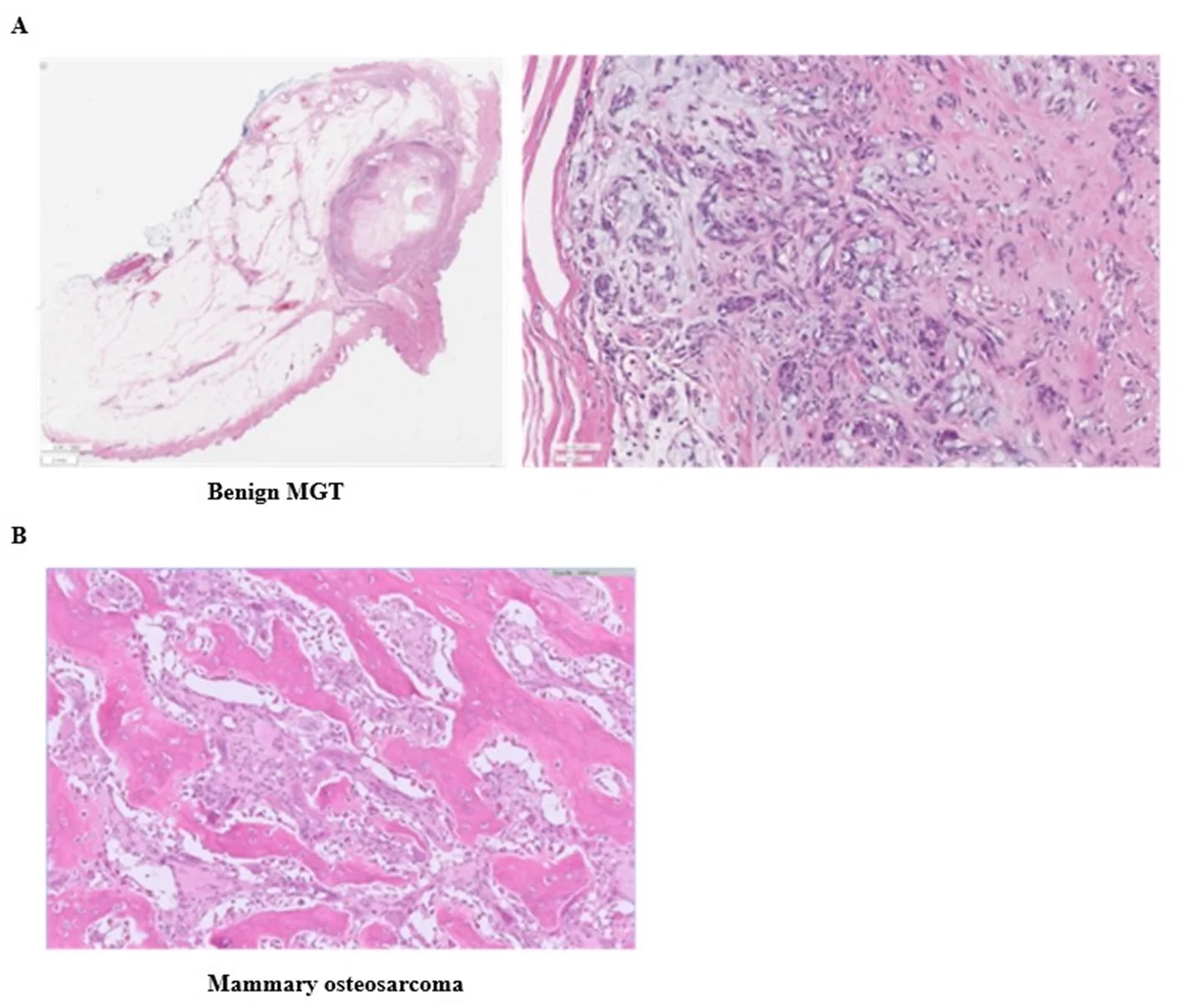
Representative histopathological images of canine mammary gland tumors (H&E stain). **(A)** Benign mixed tumor showing osseous metaplasia with well-differentiated bone formation. The epithelial foci are well differentiated and exhibit minimal atypia and no mitotic figures. **(B)** Mammary osteosarcoma characterized by malignant osteoid production and neoplastic mesenchymal cells with osteoblastic differentiation. Scale bar = 100 μm.

Histopathological examination was conducted by dividing subjects into MGT and Non-MGT groups. A group of dogs aged 2 years or younger (U2Y) was also included in the analysis to assess overall changes in microbial community composition. Including the U2Y cohort allowed us to account for age-related effects when interpreting microbial community differences, helping to distinguish tumor-associated microbial alterations from age-related variation. The diagnostic findings of dogs in the MGT group are summarized in Table 2. Dogs in the Non-MGT group were selected based on the absence of mammary neoplasia; however, several individuals presented with intercurrent non-neoplastic conditions, as detailed in Table 1, and therefore represent mammary tumor-free comparators rather than completely healthy controls. Representative hematoxylin and eosin (H&E)-stained sections illustrating the characteristic histopathological features of selected benign and malignant mammary gland tumors are shown in Figures 2A, B. Because the primary objective of this study was to investigate gut microbiome alterations associated with mammary gland tumors rather than differences among tumor subtypes, both benign and malignant tumors were analyzed together as the MGT group.

**Table 2:** Mammary gland tumor diagnosis list.

Malignancy	Sex/Age	Breed	Diagnosis
Benign	F/11 years	Poodle	Complex adenoma
	SF/11 years	Pomeranian	Complex adenoma
	SF/14 years	Maltese	Complex adenoma
	F/8 years	Cocker Spaniel	Benign mixed tumor
	F/10 years	Poodle	Benign mixed tumor
	SF/14 years	Shih Tzu	Benign mixed tumor
	SF/9 years	Jindo Dog	Complex adenoma, Benign mixed tumor
	SF/14 years	Bichon Frise	Glands, lobular hyperplasia with ductular ectasia
Malignant	SF/7 years	Poodle	Mammary carcinoma (Complex), grade 1
	F/10 years	Maltese	Mammary carcinoma (Complex), grade 1
	SF/10 years	Maltese	Mammary osteosarcoma

Hematological and biochemical parameters were compared between MGT and Non-MGT groups (Tables 3, 4). Significant differences were observed in several hematological parameters: hematocrit (HCT: 41.27 vs. 34.87%, *p* = 0.02), hemoglobin (HGB: 14.60 vs. 9.71 g/dL, *p* = 0.02), mean corpuscular hemoglobin concentration (MCHC: 32.98 vs. 26.65 g/dL, *p* = 0.01), platelet count (PLT: 527.89 vs. 294.93 × 10^9^/L, *p* = 0.03), and red blood cell count (RBC: 6.42 vs. 5.50 × 10^12^/L, *p* = 0.02). Biochemical analysis revealed significant differences in blood urea nitrogen (BUN: 26.62 vs. 17.60 mg/dL, *p* = 0.05) and BUN/creatinine ratio (BUN/CREA: 43.96 vs. 21.61, *p* = 0.01). These findings indicate that tumor-bearing dogs exhibit distinct hematological and biochemical profiles compared to controls without mammary tumors. Notably, several Non-MGT dogs showed hematological values below the normal reference range for adult female dogs, which may reflect underlying comorbidities in this group and warrants cautious interpretation of these differences.

**Table 3:** Comparison of hematological parameters between MGT and Non-MGT groups.

Parameter	MGT	Non-MGT	*p*-value
HCT (%)	41.27	34.87	0.02
HGB (g/dL)	14.60	9.71	0.02
MCH (pg)	22.66	21.61	0.60
MCHC (g/dL)	32.98	26.65	0.01
MCV (fL)	68.76	66.12	0.19
MPV (fL)	9.84	9.73	0.89
PLT (10^9^/L)	527.89	294.93	0.03
RBC (10^12^/L)	6.42	5.50	0.02
RDW-CV (%)	15.98	16.64	0.56
WBC (10^9^/L)	9.12	9.71	0.63
WBC-GRAN(#) (10^3^/mm^3^)	6.18	6.19	0.99
WBC-GRAN (%)	67.93	65.88	0.63
WBC-LYM (#) (10^9^ /L)	2.33	2.49	0.77
WBC-LYM (%)	24.89	26.65	0.67
WBC-MONO(#) (10^9^/L)	0.61	0.66	0.73
WBC-MONO (%)	7.18	6.77	0.62

**Table 4:** Comparison of biochemical parameters between MGT and Non-MGT groups.

Parameter	MGT	Non-MGT	*p*-value
GLOB (g/dL)	3.26	3.02	0.20
ALB (g/dL)	3.69	3.69	0.99
ALB/GLB	0.90	0.81	0.13
ALP (U/L)	354.00	227.83	0.27
ALT/GPT (U/L)	160.33	121.67	0.53
AST/GOT	45.67	59.50	0.60
BUN (mg/dL)	26.62	17.60	0.05
CREA (mg/dL)	0.67	0.89	0.10
BUN/CREA	43.96	21.61	0.01
GGT (U/L)	9.67	7.67	0.71
GLU (mg/dL)	114.11	103.50	0.26
PHOS (mg/dL)	3.67	4.33	0.38
TBIL (mg/dL)	0.14	0.16	0.73
T-CHOL (mg/dL)	277.50	207.44	0.11
TPRO (g/dL)	7.08	6.69	0.20
vAMY-P	940.86	739.80	0.43

The values represent the mean values of each parameter.

MGT, mammary gland tumor; GLOB, globulin; ALB, albumin; ALP, alkaline phosphatase; ALT/GPT, alanine aminotransferase; AST/GOT, aspartate aminotransferase; BUN, blood urea nitrogen; CREA, creatinine; GGT, gamma-glutamyl transferase; GLU, glucose; PHOS, phosphorus; TBIL, total bilirubin; T-CHOL, total cholesterol; TPRO, total protein; vAMY-P, pancreatic amylase.

### Gut microbiota profiling and diversity analysis via 16S rRNA sequencing

**Figure 3:**
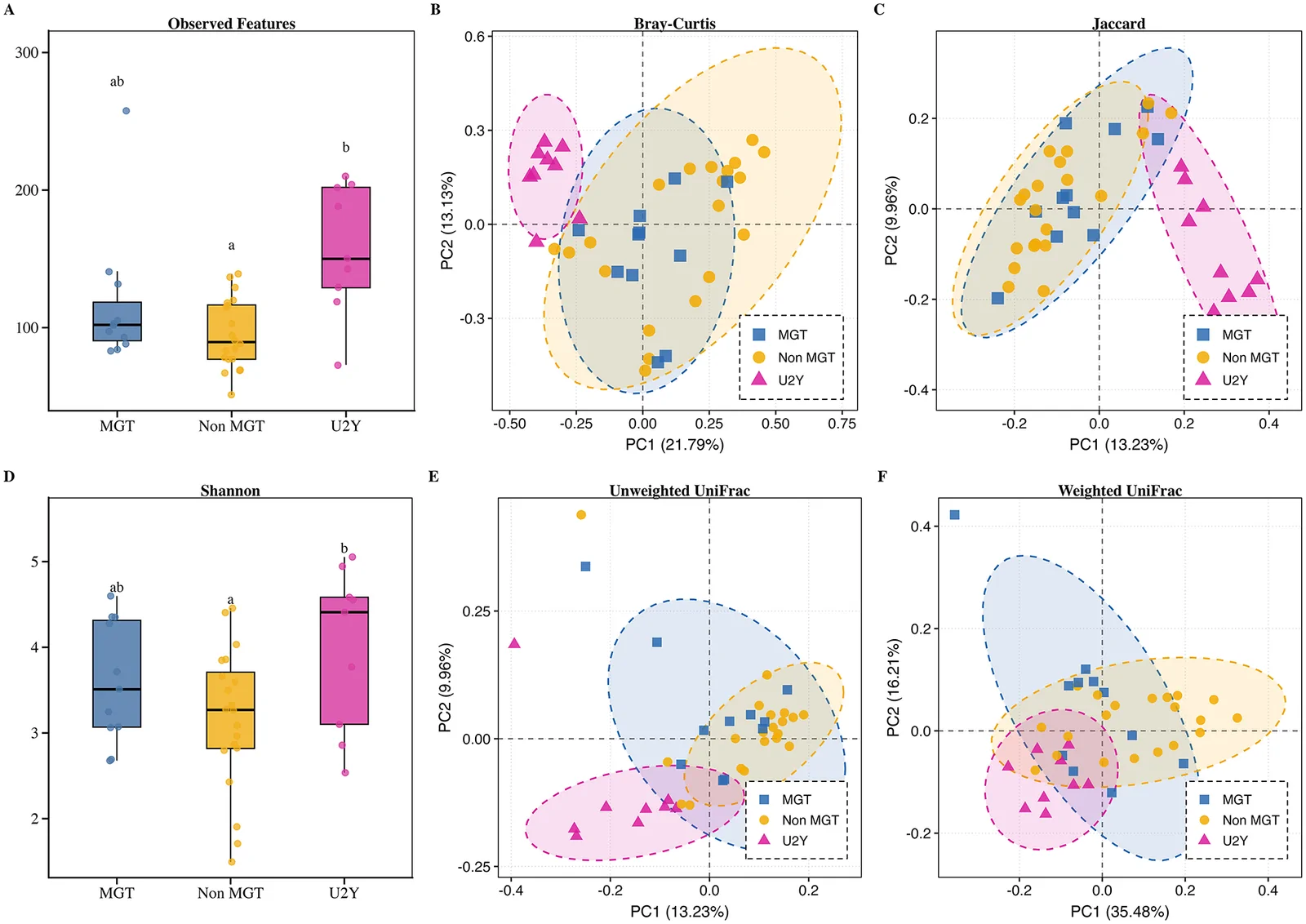
Comparison of gut microbiome diversity among MGT, Non-MGT, and U2Y groups. **(A)** Observed Features (species richness) and **(B)** Shannon Diversity Index violin plots showing alpha diversity across groups. *p*-Values were adjusted using the Benjamini–Hochberg method. **(C–F)** Principal coordinate analysis (PCoA) plots of beta diversity using Bray–Curtis, Jaccard, unweighted UniFrac, and weighted UniFrac distance metrics, respectively. PERMANOVA *p* = 0.0001 for all metrics. Each point represents one sample; colors denote group assignment.

Alpha diversity analysis revealed no significant differences between the MGT and Non-MGT groups in either species richness (Observed Features; adjusted *p* = 0.305) or Shannon diversity (adjusted *p* = 0.351). The U2Y group exhibited significantly higher species richness than the Non-MGT group (adjusted *p* < 0.001) and greater Shannon diversity (adjusted *p* = 0.047), with no significant differences observed between U2Y and MGT groups (adjusted *p* = 0.061 and 0.558, respectively; Figures 3A, B).

Beta diversity analysis via PCoA revealed significant overall differences in microbial community composition across all four distance metrics (Bray–Curtis, Jaccard, unweighted and weighted UniFrac; all PERMANOVA *p* = 0.0001). Pairwise comparisons confirmed that the U2Y group was significantly distinct from both MGT and Non-MGT groups across all metrics (adjusted *p* ≤ 0.0015). In contrast, MGT and Non-MGT groups showed no significant differences in community structure across any metric (adjusted *p* range: 0.116–0.965), indicating substantial overlap in gut microbial composition between tumor-bearing and tumor-free dogs (Figures 3C–F).

**Figure 4:**
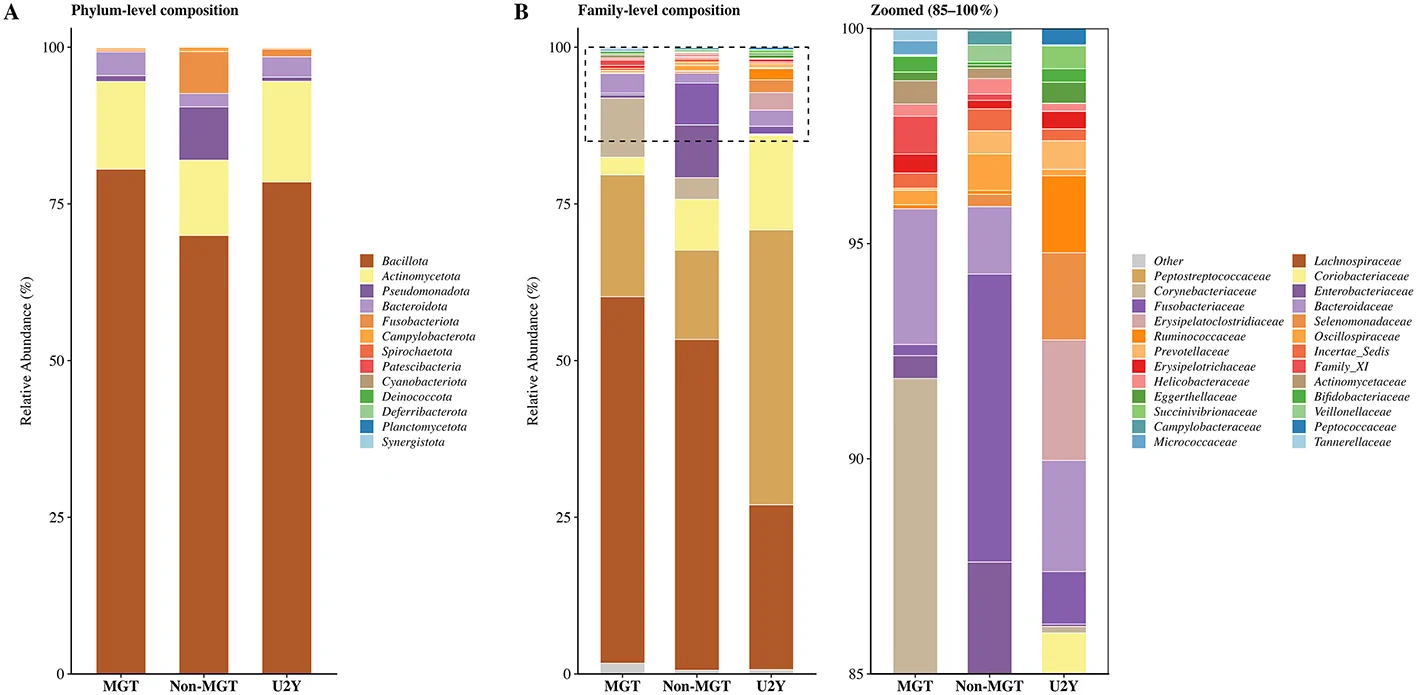
Taxonomic composition of the gut microbiota at the phylum and family levels. **(A)** Stacked bar chart showing relative abundance at the phylum level across MGT, Non-MGT, and U2Y groups. **(B)** Stacked bar chart showing relative abundance at the family level. Each bar represents an individual sample. The zoomed panel (85%−100%) highlights low-abundance families in the upper composition range. Groups are indicated above each bar set.

Consequently, we proceeded to perform a detailed comparison of microbial changes specifically between the MGT and non-MGT groups. At the phylum level, Bacillota dominated the gut microbiota across all groups, with relatively higher abundance in the U2Y and MGT groups compared with the Non-MGT group (Figure 4A). In contrast, Pseudomonadota and Fusobacteriota showed comparatively increased abundance in the Non-MGT group. Actinomycetota was consistently detected across all groups and appeared slightly enriched in the MGT group. Several minor phyla, including Fusobacteriota, Campylobacterota, Spirochaetota, and Patescibacteria, were detected at low relative abundances. These compositional differences at the phylum level are consistent with the distinct clustering patterns observed in the weighted UniFrac beta diversity analysis. Further taxonomic resolution at the family level revealed additional compositional details underlying the phylum-level differences (Figure 4B). *Lachnospiraceae* was the dominant family across all groups, with the highest relative abundance observed in the MGT and Non-MGT groups compared to U2Y. *Peptostreptococcaceae* was notably abundant in the MGT group and showed considerable inter-individual variation across adult dogs. *Corynebacteriaceae*, a family within Actinomycetota, was visibly elevated in the MGT group relative to Non-MGT and U2Y, consistent with the phylum-level trend of increased Actinomycetota in tumor-bearing dogs. *Eggerthellaceae*, the family harboring *Eggerthella*, also showed a tendency for higher abundance in the MGT group. *Coriobacteriaceae* contributed to the Actinomycetota fraction across all groups. *Enterobacteriaceae* and *Fusobacteriaceae* were present at low but detectable levels, predominantly in adult dogs. The U2Y group was distinguished by higher *Coriobacteriaceae* and *Selenomonadaceae* abundance and a notably different upper-range community composition, as evidenced in the zoomed panel (85%−100%). *Bacteroidaceae, Prevotellaceae, Ruminococcaceae*, and *Erysipelatoclostridiaceae* were present at low abundances with substantial inter-individual variation across all groups, reflecting the heterogeneous nature of canine gut microbiota. These compositional patterns provide context for the differential abundance analysis described below.

### Differential abundance analysis identifies estrobolome-associated taxa enriched in MGT

**Figure 5:**
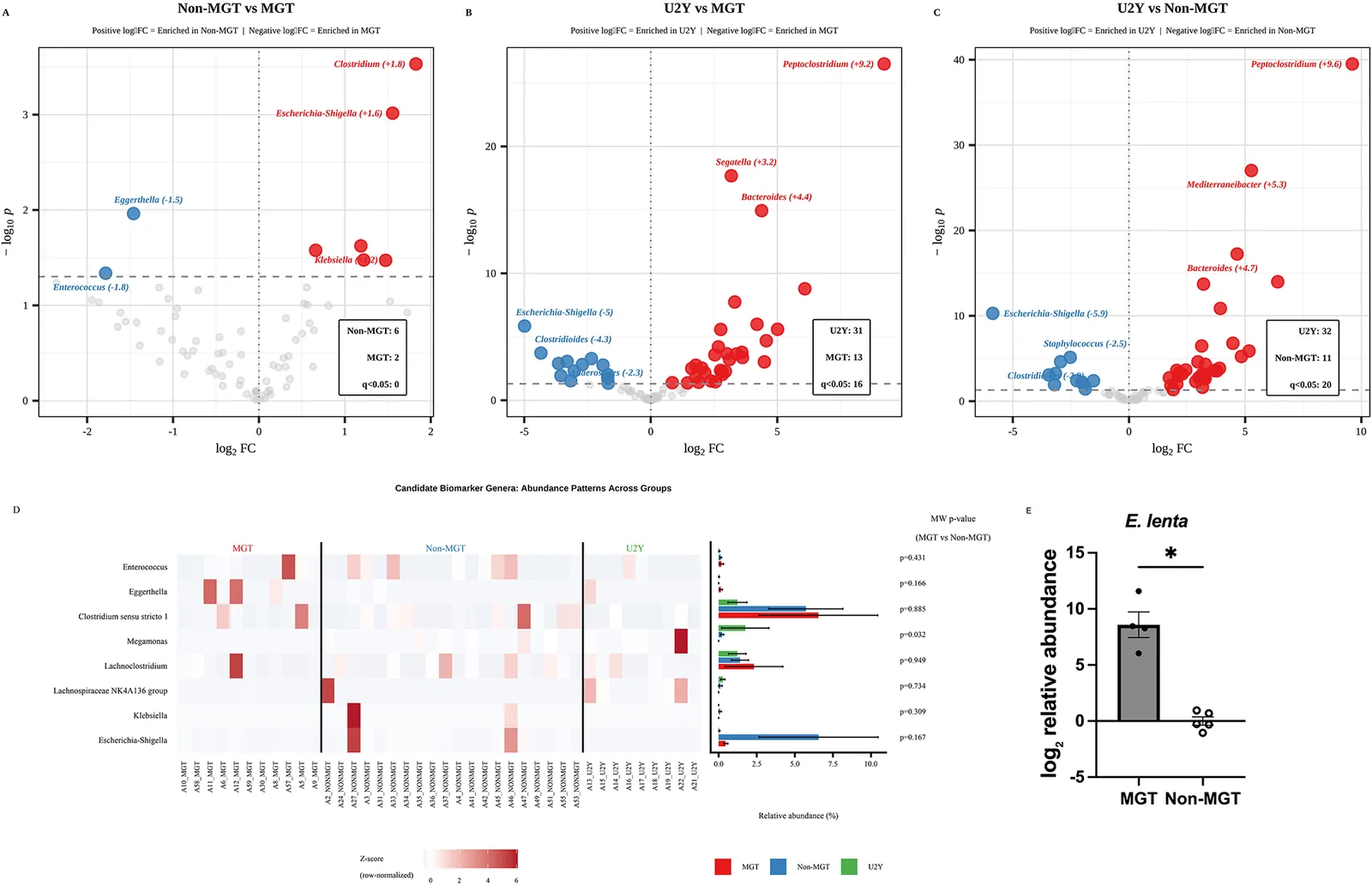
Differential abundance analysis identifies candidate microbial signatures associated with canine mammary gland tumors and validation of *Eggerthella lenta* by species-specific qPCR. **(A)** ANCOM-BC results comparing MGT vs. Non-MGT groups showing log-fold changes for significantly altered genera. **(B)** ANCOM-BC results for U2Y vs. MGT comparison. **(C)** ANCOM-BC results for U2Y vs. Non-MGT comparison. **(D)** Heatmap and relative abundance plot of candidate marker genera across individual samples. Color intensity in the heatmap reflects relative abundance; group labels are indicated above each column. The displayed Mann–Whitney *U* test *p*-values for MGT vs. Non-MGT comparisons are unadjusted exploratory *p*-values. **(E)** Validation of *Eggerthella lenta* abundance using species-specific qPCR. The bar graph shows log_2_ relative abundance, calculated using the ΔΔCt method after normalization to the universal bacterial 16S rRNA reference gene, with the mean ΔCt of the Non-MGT group used as the calibrator. *p* < 0.05 (Mann–Whitney U test)

To identify specific taxa associated with MGT status, differential abundance analysis was performed using ANCOM-BC (Figure 5). Comparison between the MGT and Non-MGT groups revealed distinct microbial signatures associated with tumor status (Figure 5A). *Eggerthella* and *Enterococcus* showed higher abundance in the MGT group (*Eggerthella*: LFC = −1.46, *p* = 0.011; *Enterococcus*: LFC = −1.78, *p* = 0.046), whereas *Clostridium, Escherichia-Shigella*, and *Klebsiella* were relatively enriched in the Non-MGT group (*Clostridium*: LFC = 1.83, *p* = 0.0003; *Escherichia-Shigella*: LFC = 1.56, *p* = 0.001; *Klebsiella*: LFC = 1.19, *p* = 0.024). No taxa survived BH-FDR correction (minimum adjusted *q* = 0.057), consistent with the limited statistical power of this small cohort (*n* = 11 MGT); these findings are therefore interpreted as directional evidence and were independently validated by species-specific qPCR. Among these taxa, *Eggerthella* is a member of the family Eggerthellaceae within Actinomycetota and is recognized as an estrobolome-associated bacterium capable of β-glucuronidase activity, which may contribute to estrogen reactivation through enterohepatic circulation. Comparisons involving the U2Y group demonstrated substantially greater microbial differences, reflecting age-associated microbiota maturation (Figures 5B, C). In the U2Y vs. MGT comparison, *Peptoclostridium, Segatella*, and *Bacteroides* were markedly enriched in the U2Y group, while *Escherichia-Shigella, Clostridioides*, and *Anaerostipes* were relatively enriched in MGT dogs. Similarly, the U2Y vs. Non-MGT comparison showed increased abundance of *Peptoclostridium, Mediterraneibacter*, and *Bacteroides* in U2Y dogs, whereas *Escherichia-Shigella, Staphylococcus*, and *Clostridioides* were more abundant in the Non-MGT group. These findings indicate that younger dogs harbor a gut microbial community composition clearly distinct from adult dogs, with age exerting a stronger influence on microbiota structure than tumor status alone. To further visualize abundance patterns of candidate marker genera, a combined heatmap and relative abundance analysis was performed (Figure 5D). The heatmap demonstrated heterogeneous but group-associated abundance distributions across individual samples. *Eggerthella* and *Enterococcus* displayed relatively higher abundance in several MGT samples, whereas *Escherichia-Shigella* and *Klebsiella* were more frequently elevated in Non-MGT dogs. *Megamonas* exhibited lower abundance in MGT compared to Non-MGT dogs by *post-hoc* Mann–Whitney *U* test on relative abundance data (*p* = 0.032); this taxon was not identified by the primary ANCOM-BC analysis and should be interpreted as a secondary, exploratory finding. The remaining genera showed non-significant but distinct distribution trends. Collectively, these results suggest that specific estrobolome- and inflammation-associated taxa may contribute to the microbial alterations observed in canine mammary gland tumors.

To validate the 16S rRNA gene sequencing results, species-specific quantitative PCR (qPCR) was performed targeting *Eggerthella lenta* in fecal DNA samples (Figure 5E). Amplification specificity was confirmed by a single melt curve peak (Supplementary Figure S1). Among the analyzed samples, *E. lenta* was detected in four of 11 MGT samples and five of 20 Non-MGT samples. Relative abundance was calculated using the ΔΔCt method after normalization to the universal bacterial 16S rRNA reference gene, with the mean ΔCt value of the Non-MGT group used as the calibrator. Despite being detected in both groups, the normalized abundance of *E. lenta* was significantly higher in the MGT group than in the Non-MGT group (Mann–Whitney *U* test, *p* = 0.0159). These results corroborate the ANCOM-BC differential abundance analysis and support the association between estrobolome bacteria and MGT status. These findings from both 16S rRNA sequencing and species-specific qPCR provide consistent evidence that estrobolome-associated *E. lenta* is significantly enriched in dogs with MGT.

### Functional pathway analysis reveals estrobolome-associated metabolic signatures in MGT

**Figure 6:**
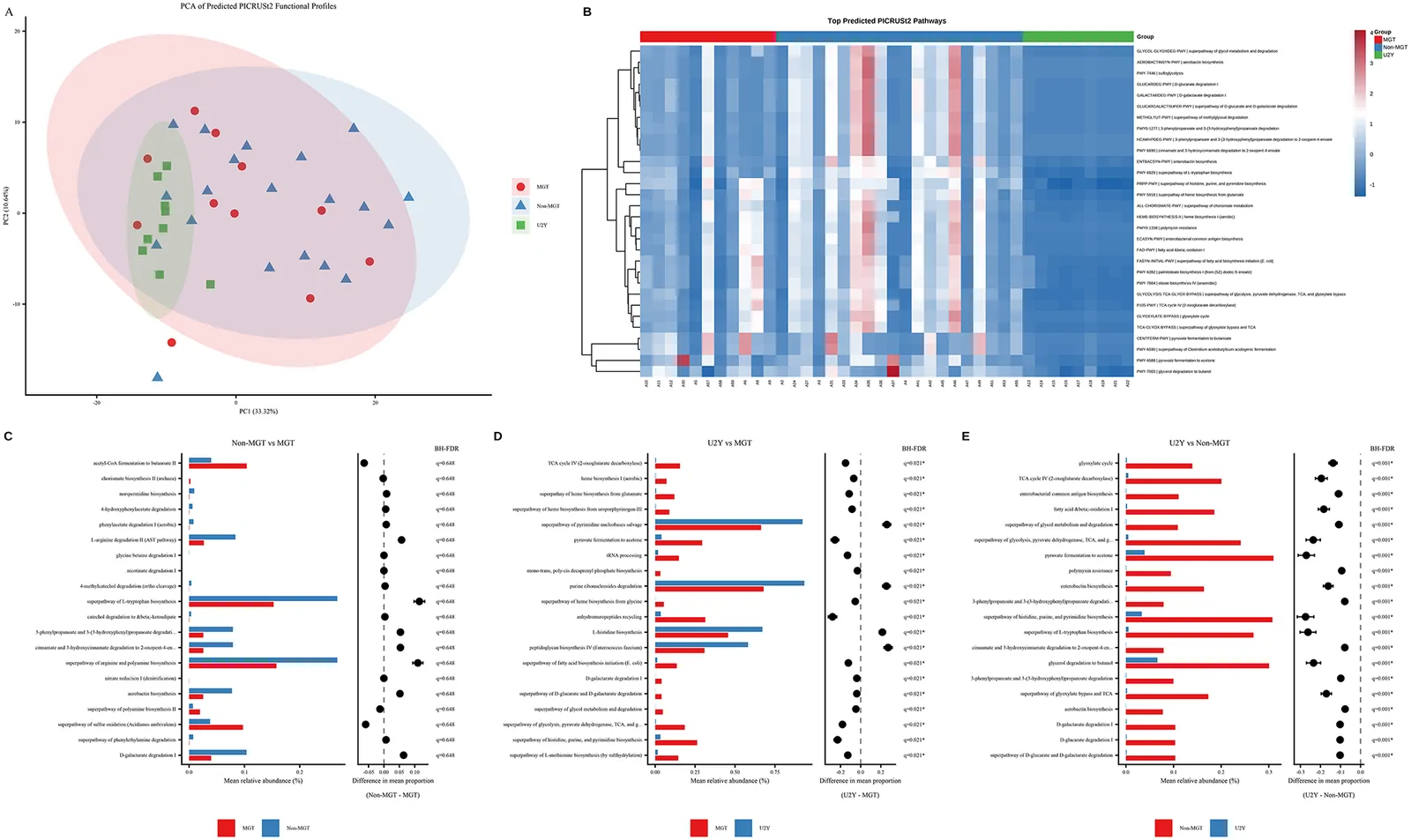
Differentially abundant functional pathways predicted by PICRUSt2. **(A)** Principal component analysis (PCA) of predicted MetaCyc pathway profiles across MGT, Non-MGT, and U2Y groups. PC1 and PC2 account for 33.32% and 10.64% of total variance, respectively. **(B)** Heatmap of top predicted MetaCyc pathways across individual samples, with group labels above columns. **(C)** Bar plot of pathways with differential relative abundance between Non-MGT and MGT groups (BH-FDR *q* = 0.648; no pathways significant after correction). **(D)** Bar plot of significantly enriched pathways in U2Y vs. MGT comparison (BH-FDR *q* = 0.021). **(E)** Bar plot of significantly enriched pathways in U2Y vs. Non-MGT comparison (BH-FDR *q* = 0.001). Error bars represent mean ± SEM. Red: MGT or Non-MGT; Blue: Non-MGT or U2Y as indicated per panel.

PICRUSt2-based functional prediction was performed to evaluate potential metabolic differences among the MGT, Non-MGT, and U2Y groups. Principal component analysis (PCA) of predicted functional profiles demonstrated substantial overlap between the MGT and Non-MGT groups, whereas the U2Y group formed a relatively distinct cluster, indicating broader age-associated functional differences in the gut microbiome (Figure 6A). PC1 and PC2 explained 33.32 and 10.64% of the total variance, respectively. Heatmap visualization of the top predicted MetaCyc pathways further demonstrated heterogeneous functional profiles across individual samples, with partial grouping according to clinical status (Figure 6B). Adult groups (MGT and Non-MGT) generally exhibited more similar pathway patterns compared with the U2Y group, consistent with the PCA results. Comparison between the Non-MGT and MGT groups identified only modest functional differences, and none of the predicted pathways remained statistically significant after BH-FDR correction (minimum *q* = 0.648; Figure 6C). Nevertheless, several pathways showed relative abundance trends between groups. The Non-MGT group demonstrated relatively higher abundance of pathways related to L-tryptophan biosynthesis, arginine and polyamine biosynthesis, aromatic compound degradation, and D-galactarate degradation, whereas the MGT group showed relatively higher abundance of acetyl-CoA fermentation to butanoate II, superpathway of polyamine biosynthesis II and sulfur oxidation pathways. In contrast, comparisons involving the U2Y group revealed more pronounced functional differences. In the U2Y vs. MGT comparison, several pathways associated with nucleotide metabolism, amino acid biosynthesis, and peptidoglycan biosynthesis showed significantly higher relative abundance in the U2Y group following BH-FDR correction (*q* = 0.021; Figure 6D). These included pathways involved in pyrimidine nucleobase salvage, purine ribonucleoside degradation, L-histidine biosynthesis, and peptidoglycan biosynthesis IV. Conversely, pathways related to heme biosynthesis, pyruvate fermentation to acetone, and glycol-related metabolism showed relatively higher abundance in the MGT group. Similarly, the U2Y vs. Non-MGT comparison demonstrated significant enrichment of multiple pathways in the Non-MGT group following BH-FDR correction (*q* = 0.001; Figure 6E). These included pathways associated with glycolysis and TCA-linked metabolism, glyoxylate cycle activity, enterobactin biosynthesis, pyruvate fermentation, aromatic compound degradation, and L-tryptophan biosynthesis. The U2Y group showed comparatively lower abundance across many of these pathways. Overall, these findings suggest that age exerts a stronger influence on predicted microbial functional profiles than tumor status alone. Although several metabolic pathway trends were observed between MGT and Non-MGT groups, the substantial functional overlap and lack of statistically significant differences after multiple-testing correction indicate that these predicted functional changes should be interpreted cautiously.

## Discussion

The present study investigated the gut microbiota composition of female dogs with mammary gland tumors (MGTs) and compared it with age-matched mammary tumor-free adult dogs (Non-MGT), acknowledging that this group included dogs with intercurrent non-neoplastic comorbidities and thus represents a clinical comparator rather than a strictly healthy control population, and young dogs under 2 years of age (U2Y) using 16S rRNA gene sequencing and PICRUSt2 functional prediction. Our findings suggest a pattern of gut microbial dysbiosis in MGT-bearing dogs, characterized by enrichment of the estrobolome-associated bacterium *Eggerthella* and concurrent depletion of SCFA-producing genera. PICRUSt2 functional prediction further suggested trends toward increased polyamine biosynthesis and acetyl-CoA fermentation to butanoate II in MGT dogs compared with Non-MGT controls, whereas pathways related to heme biosynthesis and central carbon metabolism were significantly enriched in the comparison between MGT and U2Y dogs. Collectively, these results suggest a gut microbial environment that may promote estrogen-driven mammary tumorigenesis through enhanced enterohepatic estrogen recycling, metabolic reprogramming, and reduced anti-tumor immune capacity.

Alpha diversity indices showed no significant differences between the MGT and Non-MGT groups in species richness or Shannon diversity, a pattern consistent with human breast cancer microbiome studies in which compositional shifts occur in the absence of overall diversity loss (Yang et al., 2021; Chen et al., 2025). This suggests that MGT-associated dysbiosis manifests as a qualitative restructuring of the microbial community rather than a reduction in total species number. Notably, the U2Y group exhibited significantly higher species richness and Shannon diversity compared to the Non-MGT group, reflecting the ongoing process of microbiota maturation in young animals, wherein communities remain dynamically diverse before stabilizing in adulthood (Aluai-Cunha et al., 2023). Beta diversity analysis confirmed that the U2Y group was compositionally distinct from both adult groups across all four-distance metrics. Although MGT and Non-MGT groups showed substantial overlap in overall community structure after correction for multiple comparisons, differential abundance analysis identified specific taxa that distinguished tumor-bearing from tumor-free dogs, indicating that tumor-associated microbiota differences are primarily driven by shifts in the relative abundance of specific taxa rather than the presence or absence of unique species.

The central finding of this study is the significant enrichment of *Eggerthella* in MGT dogs, confirmed at species resolution by qPCR demonstrating significantly higher normalized *E. lenta* abundance in MGT samples (Mann–Whitney *U* test, *p* = 0.0159). *E. lenta* is a Gram-positive anaerobe within Eggerthellaceae that encodes a characterized beta-glucuronidase capable of deconjugating glucuronidated estrogen metabolites in the intestinal lumen, thereby reactivating free estrogens for enterohepatic reabsorption (Ervin et al., 2019; Hu et al., 2023; Tsai et al., 2025). This enzymatic activity may partially counteract hepatic phase II glucuronidation, a primary estrogen detoxification pathway, and can substantially elevate circulating estradiol and estrone concentrations (Plottel and Blaser, 2011; Flores et al., 2012). The relevance of this mechanism to mammary tumorigenesis is well established in human breast cancer: elevated urinary estrogen-to-glucuronide ratios, indicative of enhanced deconjugation, have been associated with increased breast cancer risk (Tsai et al., 2025), and *Eggerthella* and related *Coriobacteriaceae* have been repeatedly identified in breast cancer-associated microbiome studies (Xuan et al., 2014; Luu et al., 2017; Parida and Sharma, 2019). Given that approximately 88% of malignant canine mammary tumors are hormone receptor-positive (Gautam et al., 2025), the enrichment of *E. lenta* in MGT dogs is mechanistically plausible and consistent with the elevated estrogen milieu implicated in tumor proliferation. To our knowledge, this is among the first studies to quantify *E. lenta* abundance in canine MGT, and the qPCR validation provides additional support for the differential abundance signal initially identified by 16S rRNA sequencing.

Complementing the enrichment of pro-estrogenic taxa, we observed a trend toward reduced *Megamonas* abundance in MGT dogs (*post-hoc* Mann–Whitney *p* = 0.032; not identified by primary ANCOM-BC analysis) and a trend toward reduced *Clostridium* abundance in MGT dogs. These genera are commonly associated with short-chain fatty acid (SCFA) production, particularly butyrate and propionate, which have been linked to anti-inflammatory and anti-tumor effects in previous studies (Koh et al., 2016; Al-Qadami et al., 2022; Fusco et al., 2023; Du et al., 2024). Butyrate inhibits histone deacetylases (HDACs), suppressing the expression of oncogenic targets and promoting apoptosis in transformed epithelial cells (Donohoe et al., 2012; Nepelska et al., 2012). At the systemic level, SCFAs suppress NF-κB-driven pro-inflammatory and pro-tumorigenic signaling and support cytotoxic immune effector activity, representing important mechanisms of gut-distal anti-tumor immune modulation (Smith et al., 2013; Kim, 2014; Tan et al., 2014). Depletion of these taxa in tumor-bearing dogs is consistent with reports of reduced SCFA-producing bacteria in human breast cancer patients (Ma et al., 2018) and suggests that the dysbiotic shift in canine MGT may simultaneously enhance estrogenic drive and erode SCFA-dependent anti-tumor immune surveillance. The concurrent enrichment of *Enterococcus* in the MGT group warrants attention, as certain *Enterococcus* species produce deconjugases with overlapping estrogen-reactivating activity, and *Enterococcus faecalis* has been linked to reactive oxygen species-mediated DNA damage in colonic epithelium (Huycke et al., 2001, 2002; Wang et al., 2008).

PICRUSt2 functional pathway analysis provided additional mechanistic context for the observed microbial dysbiosis. Although no individual pathway remained statistically significant after BH-FDR correction in the direct comparison between MGT and Non-MGT dogs (Figure 6C), several biologically relevant trends were observed. Compared with Non-MGT dogs, the MGT group showed a trend toward higher predicted abundance of both the superpathway of polyamine biosynthesis II and the acetyl-CoA fermentation to butanoate II pathway. Polyamines such as spermidine and spermine are well-established promoters of tumor cell proliferation; they stabilize DNA and RNA structures, facilitate ribosome biogenesis, and activate oncogenic signaling cascades, and their biosynthesis is upregulated in breast cancer tissue and linked to poor prognosis (Akinyele and Wallace, 2021, 2022; Xuan et al., 2023). Although the predicted acetyl-CoA fermentation to butanoate II pathway was relatively enriched in MGT dogs, this finding contrasts with the observed depletion of putative butyrate-producing genera, including *Megamonas* and *Clostridium*. Since PICRUSt2 predicts functional potential rather than directly measuring metabolic activity, these findings should be interpreted cautiously and require validation using metabolomic or shotgun metagenomic approaches (Donohoe et al., 2012; Smith et al., 2013). In the U2Y vs. MGT comparison (Figure 6D), MGT dogs exhibited significantly higher predicted abundance of heme biosynthesis pathways (*q* = 0.021). Heme and its metabolic intermediates can serve as substrates for peroxidase reactions that generate reactive oxygen species, contributing to oxidative DNA damage in epithelial cells and promoting a pro-tumorigenic microenvironment (Seiwert et al., 2020; Consoli et al., 2025). Additionally, pyruvate fermentation to acetone and other central carbon metabolism pathways were enriched in MGT relative to U2Y, consistent with a metabolic environment associated with active fermentation and energy production that may parallel the metabolic reprogramming observed in tumor cells (Fukushi et al., 2022). Taken together, these predicted functional alterations may reflect microbial metabolic changes associated with canine MGTs and provide biologically plausible hypotheses linking microbial dysbiosis to estrogen metabolism and tumor-related pathways. However, these predictions are inferential and do not demonstrate activation of host signaling pathways; validation using shotgun metagenomics, metatranscriptomics, or targeted metabolomics will be required to confirm these hypothesis-generating functional signatures (Douglas et al., 2020).

From a translational perspective, the identification of *E. lenta* as a significantly enriched and qPCR-validated taxon in MGT-bearing dogs suggests that it may represent a candidate non-invasive fecal microbial marker for canine mammary gland tumors. Importantly, *E. lenta* was detected in both MGT and Non-MGT dogs, indicating that its potential value may lie in differences in relative abundance rather than simple presence or absence. Similar abundance-based microbial signatures have been reported in other microbiome studies, where quantitative differences rather than exclusive detection distinguish disease from control groups (Duvallet et al., 2017). In the present study, species-specific qPCR demonstrated significantly higher normalized relative abundance of *E. lenta* in MGT dogs, consistent with the 16S rRNA sequencing results. These findings provide independent support for the enrichment of *E. lenta* in canine MGT but should be interpreted cautiously given the limited sample size. Further validation in larger, independent cohorts will be necessary to determine whether *E. lenta* has potential clinical utility as a microbial biomarker and to establish clinically relevant abundance thresholds, diagnostic sensitivity, specificity, and predictive value before clinical application. Current diagnostic approaches for MGTs rely on clinical palpation, fine needle aspiration, and histopathological assessment, which are inherently invasive, may miss early-stage lesions, and are subject to sampling variability (Kaszak et al., 2022). In this context, the observed enrichment of *E. lenta* suggests that this organism merits further investigation as a candidate component of microbiome-based approaches for canine MGT. The biological relevance of *E. lenta* is supported by its established role in estrogen metabolism and the plausibility that alterations in the estrobolome may influence hormone-dependent mammary tumorigenesis (Plottel and Blaser, 2011; Parida and Sharma, 2019). Moreover, a multi-marker fecal panel combining *E. lenta* abundance with depletion indices for *Megamonas* and *Clostridium* could potentially improve the discriminatory power of a gut microbiome-based diagnostic approach for MGT. Such a panel could potentially capture both the pro-estrogenic and potentially protective components of the dysbiotic profile identified in the present study, and would require prospective evaluation before it could be considered for development into a cost-effective assay for veterinary clinical settings. Validation of *E. lenta* as a candidate diagnostic marker in larger prospective cohorts, with correlation to serum estrogen metabolite profiling and tumor hormone receptor status, represents a critical next step and a priority for future investigation (Kaszak et al., 2022; Larnder et al., 2025).

The present study should be regarded as a pilot investigation, and several limitations should be acknowledged. The relatively small sample sizes, particularly in the MGT (*n* = 11) and U2Y (*n* = 9) groups, may limit statistical power and generalizability of findings. In addition, *E. lenta* was detected by species-specific qPCR in only a subset of animals in each group (four of 11 MGT and five of 20 Non-MGT samples), so the quantitative comparison of amplification data was restricted to amplification-positive samples; the magnitude of the observed difference in abundance therefore requires confirmation in larger cohorts using assays with greater analytical sensitivity. The heterogeneity in MGT histological subtypes (benign vs. malignant) and the absence of hormone receptor status data for individual tumors precluded subgroup analyses that could clarify whether the estrobolome dysbiosis is more pronounced in receptor-positive cases. Additionally, as this is a cross-sectional observational study, causality between *E. lenta* enrichment and tumor development cannot be established; it is possible that tumor-induced systemic changes alter the intestinal environment in ways that selectively favor *E. lenta* colonization as a consequence rather than a cause of tumorigenesis. The functional predictions generated by PICRUSt2 are based on reference genome databases and may not fully capture the metabolic repertoire of the canine gut microbiome; confirmation by shotgun metagenomics and targeted metabolomics is warranted. Finally, dietary information, reproductive history, and body condition scores were not systematically collected for all subjects, each of which can independently influence gut microbiome composition and estrogen metabolism. Despite these limitations, the present study provides the first species-level evidence of an association between *E. lenta* and canine mammary gland tumors and establishes a foundation for future research. Longitudinal studies integrating shotgun metagenomics, metabolomics, hormonal profiling, and experimental models such as germ-free or gnotobiotic animals will be important to determine whether the observed microbial alterations contribute causally to canine mammary tumorigenesis and to evaluate their potential as microbiome-based diagnostic or therapeutic targets.

## Conclusion

In conclusion, this pilot study indicates that gut microbial dysbiosis in canine mammary gland tumors is characterized by enrichment of the estrobolome-associated bacterium *Eggerthella lenta*, depletion of short-chain fatty acid-producing bacteria, and predicted functional alterations involving polyamine biosynthesis, heme biosynthesis, and glycolytic pathways. Together, these findings provide evidence for a potential association between the gut microbiome, estrogen metabolism, and mammary tumor biology. Importantly, *E. lenta* was identified as a candidate fecal microbial marker that warrants validation in larger, independent prospective cohorts before clinical application. Overall, this study provides new insights into the gut–mammary axis and supports future investigations exploring microbiome-based diagnostic and therapeutic strategies in canine mammary gland tumors.

## Data Availability

The data presented in this study are associated with BioProject accession KAP242401, and the corresponding Korea Sequence Read Archive (KRA) accession is KRA2463028. The BioProject KAP242401 and all 40 sequencing datasets associated with KRA2463028 are publicly accessible through K-BDS/KRA: https://kbds.re.kr/search/total?keyword=KRA2463028.
