# Supplementary material for: Estrobolome dysbiosis in canine mammary gland tumors: Eggerthella lenta as a candidate microbial marker for hormone-dependent mammary tumorigenesis
Source: Front Microbiol. 2026 Sep 2;17:1923235. doi: 10.3389/fmicb.2026.1923235 (PMC13580333; doi:10.3389/fmicb.2026.1923235)
Supplement: Supplementary file 1 [file Supplementary_File_1.docx]

**Estrobolome dysbiosis in canine mammary gland tumors: *Eggerthella lenta* as a candidate microbial marker for hormone-dependent mammary tumorigenesis**

Kun Woo Kim^1, 2,^ †, Mahnoor Farooq^3, 4,^ †, Do Young Jin^5,^ †, Wonhong Kim^3^, Jiho Shin^3, 4^, Suji Park^3, 4^, Ginseo Ko^3^, Seohyeon Park^3^, Gyeongtae Kim^3^, Seunghyun Na^3^, Minsoo Kim^3^, Yae-ji Kim^3, 4^, Chaehyun Kim^5^, Ho Jun Lee^2^, Sungyoung Choi^1, 2^, Hyo-Jin Ahn^6^, Hyuk-Ju Jang^7^, Han-Sol Choi^8^, Ki-Young Yoon^9^, Kyung-Ku Kang^10^, Suk-Chae Jung^11^, Woo Jin Song^12^, Jun-Seob Kim^3,^ *, Young Hyun Jung^3, 4^ *

^1^Laboratory of Veterinary Internal Medicine, College of Veterinary Medicine, Jeju National University, Jeju 63243, Korea

^2^FM Animal Medical Center, Goyang-si, Gyeonggi-do, Republic of Korea

^3^Department of Physiology, College of Medicine, Soonchunhyang University, Cheonan, 31151, Republic of Korea

^4^Institute for Molecular Metabolism Innovation, Soonchunhyang University, Asan 31538, Republic of Korea

^5^Department of Nano-Bioengineering, Incheon National University, Incheon, Republic of Korea

^6^New S Animal Hospital, Gunpo-si, Gyeonggi-do, Republic of Korea

^7^Able Animal Medical Center, Seocho-gu, Seoul, Republic of Korea

^8^Department of Veterinary Nursing, Shingu College, Seongnam-si, Gyeonggi-do, Republic of Korea

^9^Department of Companion Animal, Shingu College, Seongnam-si, Gyeonggi-do, Republic of Korea

^10^Preclinical Research Center, K-MEDI hub, Daegu, Korea

^11^Department of Chemical and Biomolecular Engineering, University of Illinois at Urbana-Champaign, Urbana, IL, 61801, USA

^12^Signature Animal Medical Center, Seoul, Republic of Korea.

†These authors have contributed equally to this work and share first authorship

*Corresponding author: [yhjung@sch.ac.kr](mailto:yhjung@sch.ac.kr) (YHJ), [junkim@inu.ac.kr](mailto:junkim@inu.ac.kr) (JSK)

**Supplementary Figure S1. Representative qPCR amplification and melt curve analysis for *Eggerthella lenta*.**

**(A)** Amplification curves, MGT samples.

**(B)** Melt peak curves, MGT samples.

**(C)** Amplification curves, Non-MGT samples.

**(D)** Melt peak curves, Non-MGT samples.

**(E)** Amplification curves, MGT (red) and Non-MGT (blue) overlaid.

**(F)** Melt peak curves, MGT (red) and Non-MGT (blue) overlaid.

Representative amplification curves (A, C, E) and melt curve analyses (B, D, F) from the species-specific qPCR assay for *Eggerthella lenta* in canine fecal DNA. Panels A and B show MGT samples, panels C and D show Non-MGT samples, and panels E and F show both groups overlaid (red, MGT; blue, Non-MGT). The horizontal line indicates the detection threshold. Melt curve analysis demonstrated a consistent single dominant melting peak in all amplification-positive samples, supporting the specificity of the assay.
